# Changes in the vitamin D endocrine system and bone turnover after oral vitamin D3 supplementation in healthy adults: results of a randomised trial

**DOI:** 10.1186/1472-6823-12-7

**Published:** 2012-06-13

**Authors:** Kristin Holvik, Ahmed A Madar, Haakon E Meyer, Cathrine M Lofthus, Lars C Stene

**Affiliations:** 1 Division of Epidemiology, Norwegian Institute of Public Health, Oslo, Norway; 2 Institute of Health and Society, University of Oslo, Oslo, Norway; 3 Department of Endocrinology, Oslo University Hospital, Oslo, Norway; 4 Norwegian Institute of Public Health, P.O. Box 4404, Nydalen, 0403, Oslo, Norway

**Keywords:** Supplementation, Randomised trial, Vitamin D3, Cholecalciferol, 25-hydroxyvitamin D, 1,25-dihydroxyvitamin D, Parathyroid hormone, Tartrate-resistant acid phosphatase, Bone turnover

## Abstract

**Background:**

There is uncertainty as to which intake of vitamin D is needed to suppress PTH and maintain normal bone metabolism throughout winter at northern latitudes. We aimed to investigate whether four weeks’ daily supplementation with 10 μg vitamin D3 from fish oil produced a greater change in serum vitamin D metabolites, parathyroid hormone, and bone turnover in healthy adults compared with solid multivitamin tablets. Furthermore, it was studied whether age, gender, ethnic background, body mass index, or serum concentrations at baseline predicted the magnitude of change in these parameters.

**Methods:**

Healthy adults aged 19–48 years living in Oslo, Norway (59°N) were randomised to receive a daily dose of 10 μg vitamin D3 given as fish oil capsules or multivitamin tablets during four weeks in late winter. Serum samples from baseline and after 28 days were analysed for 25-hydroxyvitamin D (s-25(OH)D), 1,25-dihydroxyvitamin D (s-1,25(OH)_2_D), intact parathyroid hormone (s-iPTH), and osteoclast-specific tartrate-resistant acid phosphatase 5b (s-TRACP). Fifty-five eligible participants completed the intervention (74% of those randomised).

**Results:**

S-25(OH)D increased by mean 34.1 (SD 13.1) nmol/l, p < 0.001; s-iPTH decreased by mean 1.2 (SD 2.5) pmol/l, p = 0.001; s-1,25(OH)_2_D increased by mean 13 (SD 48) pmol/l, p = 0.057; and s-TRACP increased by mean 0.38 (SD 0.33) U/l, p < 0.001. For all these parameters, there was no difference between fish oil and multivitamin formulation. Baseline concentrations were the only independent predictors of changes in biochemical parameters.

**Conclusions:**

Four weeks of daily supplementation with 10 μg vitamin D3 decreased mean s-iPTH and increased s-TRACP concentration, and this did not differ by mode of administration. Our results suggest an increased bone resorption following vitamin D supplementation in young individuals, despite a decrease in parathyroid hormone levels.

**Trial Registration:**

ClinicalTrials.gov: NCT01482689

## Background

Low vitamin D status is commonly observed at northern latitudes such as in Scandinavia during wintertime, with a drop in serum concentrations of 25-hydroxyvitamin D (s-25(OH)D) and a corresponding increase in serum parathyroid hormone levels (PTH) and bone turnover [1,2]. In addition to latitude and season, individual sun exposure and vitamin D supplementation are strong predictors of vitamin D status [1]. Maintaining s-25(OH)D and suppressing PTH through winter is expected to be beneficial in order to prevent bone resorption. Possible direct means of increasing wintertime vitamin D status at northern latitudes may be increased fortification of foods [3], or increased supplementation. Current recommended dietary intake of vitamin D for adults in European countries and the US varies between 0–15 μg/day (20 μg/day for elderly in the US) [4,5]. Daily supplementation with 10 μg (400 IU) vitamin D is recommended to elderly individuals with low sun exposure in the Nordic countries [6]. However, there is uncertainty as to which supplemental dose is needed in order to maintain 25(OH)D levels associated with PTH suppression and normal bone metabolism. In an experimental study performed in Omaha (41°N) it was estimated that daily oral supplementation with 12.5 μg (500 IU) may be required in order to maintain autumn s-25(OH)D levels of 70 nmol/l through winter [7]. Daily oral supplementation with 10 μg (400 IU) or 20 μg (800 IU) vitamin D3 prevented increase in PTH and bone turnover during winter in healthy men in Helsinki, Finland (60°N) (n = 48) [8]. There seems to be large variation in s-25(OH)D in response to oral supplementation. According to a summary of various studies published up to 1999 [9], a daily dose of 10 μg (400 IU) vitamin D led to an average increase in s-25(OH)D of 31 nmol/l, although with large variation between studies, suggesting that additional factors may influence the degree of increase. A systematic review concluded that the degree of PTH suppression achieved by vitamin D supplementation depends not only on baseline PTH and increase in s-25(OH)D, but also on the subjects’ age and mobility [10]. Healthy young students in Coleraine, Northern Ireland (55°N) who received a daily oral supplement of 15 μg (600 IU) vitamin D3 for 8 weeks in late winter, increased their mean s-25(OH)D from 47.9 to 86.5 nmol/l, but the supplementation did not influence PTH levels or bone turnover significantly [11]. In a recent 2-center double-blind placebo-controlled study from the same group, 215 persons aged 20–40 received 0, 5, 10, or 15 μg vitamin D3 daily during 22 weeks in late winter, but supplementation did not affect PTH levels or bone turnover [12]. Elderly osteoporotic women in sunshine abundant São Paulo, Brazil (23°S) who received 10 μg (400 IU) vitamin D3 daily for three months increased their mean s-25(OH)D from 46.7 to 59.5 nmol/l, whereas PTH levels or bone turnover were not significantly affected [13].

In our experimental study performed in Oslo (59°N) during late winter, we found a similar effect of fish oil capsules and multivitamin D tablets on increase in s-25(OH)D [14]. We here present results for the predefined secondary objectives of the study: (a) to compare changes in s-1,25(OH)_2_D, s-iPTH and s-TRACP in the fish oil capsule vs. multivitamin tablet groups, and (b) in the supplement groups combined, to investigate whether the magnitude of change in s-25(OH)D and the above mentioned metabolites was predicted by ethnic background, body mass index, age, gender, or serum concentrations at baseline.

## Methods

### Study subjects

Subjects with Norwegian (in the following quoted as Norwegians) and other ethnic background were recruited primarily among medical students and nurse students in Oslo. In addition, subjects with Sri Lankan Tamil background (in the following quoted as Tamils) were recruited through an organisation for Tamils in Oslo (Tamil Resource and Counselling Centre). The participants recruited through medical or nursing school attended pre-test baseline examination within the period 14–17 February, and the participants recruited through the Tamil Resource and Counselling Centre attended within the period 11–14 March. The four-week intervention was thus completed in mid-April by the latest, i.e. prior to Easter holidays when sunlight exposure would be expected to increase. Those who already took a vitamin D supplement regularly, defined as once a week or more, or had been travelling to sunny areas or used a tanning bed during the previous three months, were defined as ineligible to participate. A total of 74 subjects of the 143 subjects who had agreed to participate (51.7%), were randomised to receive multivitamin tablets or fish oil capsules [14].

### Intervention

The participants each received a daily supplement of 10 μg (400 IU) vitamin D3 from one multivitamin tablet of type Vitaplex ABCD (Cederroth AS, Revetal, Norway), or one fish oil capsule, specially manufactured for this study by Peter Möller (now Möller’s, Oslo, Norway). Vitamin D3 content of the supplements was determined by an independent laboratory (AS Vitas, Oslo, Norway). Mean content was 9.79 μg per multivitamin tablet (SD 1.51), and 9.99 μg per fish oil capsule (SD 0.23), respectively. In addition to vitamin D3, the multivitamin tablets were declared by the manufacturer to contain 750 μg vitamin A, 1.5 mg vitamin B1, 1.7 mg vitamin B2, 1.5 mg vitamin B6, 20 mg niacin, 5 mg pantothenic acid, and 30 mg vitamin C. The fish oil capsules contained 660 μg vitamin A, 3.7 mg vitamin E, and 0.5 g n-3 polyunsaturated fatty acids, as analysed by the manufacturer. Retinol concentration in the fish oil capsules was standardised to be similar to the multivitamin tablets. Supplements were handed out to each participant at baseline, along with a self-administered compliance form. Participants were encouraged to maintain their usual diet during the study period. The tablets or capsules were ingested daily for 4 weeks, which is considered to be a sufficient duration in order to reach equilibrium (plateau concentration) in s-25(OH)D [9].

### Data collection

A venous serum sample was drawn on day 1, prior to the intervention, and on day 29, the day subsequent to ingesting the final tablet or capsule. The participants completed a self-administered three-page questionnaire at baseline. During completion of the questionnaire, they had the possibility to ask for assistance (i.e. clarification of questions, or language issues) from one of the project leaders. The questionnaire included questions about usual intake of vitamin D-containing foods, supplement use, clothing and sun exposure habits, self-reported height and weight, date of birth, education, and ethnic background. At follow-up, all participants’ height and weight were measured with an electronic height- and weight-measuring device.

### Serum sample handling and analyses

Blood samples were centrifuged (10 min, 2000 g at 10 °C) within 30 minutes after blood collection and sera were immediately frozen and kept at −70 °C until analyzed. Pre- and post-intervention serum from all participants were analysed blinded, in one batch at the Hormone Laboratory (Department of Endocrinology, Oslo University Hospital Aker, Oslo, Norway).

S-25(OH)D was measured by radioimmunoassay (RIA) (DiaSorin, Stillwater, MN, USA). This assay measures both 25(OH)D_3_ and 25(OH)D_2._ The intra- and interassay coefficients of variation (CV) were 6% and 14-15%, respectively. The detection limit was 6 nmol/l.

Intact PTH (iPTH) were measured by chemiluminoimmunometric assay (DPC, Los Angeles, CA, USA). The intra- and interassay CVs for iPTH were 4% and 10%, respectively.

S-1,25(OH)_2_D was measured by competitive RIA (DiaSorin, Stillwater, MN, USA). Prior to the 1,25(OH)_2_D determination, serum lipids and interfering vitamin D metabolites were removed by chromatography on a C18OH column. Cross reaction with 25(OH)D after chromatography is noted to be 0.002%. The intra- and interassay CVs for the s-1,25(OH)_2_D assay were 7 and 14%, respectively. The limit of detection was 12 pmol/l.

S-TRACP was measured by enzyme activity assessment after immune extraction (Suomen Bioanalytiikka Oy, Oulu, Finland). This assay measures the active isoform 5b derived from osteoclasts. The intra- and interassay CVs for s-TRACP were 5–12 and 8–14% respectively.

### Statistical analysis

The data were analysed in SPSS and Stata. According to normality plots, the dependent variables: Δ25(OH)D, Δ PTH, Δ1,25(OH)_2_D and ΔTRACP did not deviate substantially from a normal distribution, and non-parametric tests yielded similar results. Therefore, means and distributions are presented for continuous variables, and percentages are presented for categorical variables. Pre- and post-intervention concentration of biochemical parameters were compared by paired samples t tests. Crude differences between the two intervention groups were tested using *t* test. Potential predictors of change in biochemical parameters were analysed using linear regression.

## Results

### Participants

Of the 74 subjects included in the study, 10 violated the eligibility criteria according to the questionnaire at baseline, three reported an intake of less than 26 tablets or capsules during the 28-day intervention period, three were lost to follow-up, two had their second blood sample taken three days before schedule, and one withdrew. The number of participants excluded was similar in the two intervention groups (nine and ten, respectively). Thus, 55 eligible participants completed the study. Of these, 36 had ethnic Norwegian background, 12 had Tamil background, and seven had various other ethnic backgrounds. Characteristics of the study participants are presented in Table 1.

**Table 1:** Background characteristics of participants (n = 55)

Age, years, mean (range)	28 (19–48)
Gender, n (%) women	35 (63.6)
Body mass index, kg/m^2^, mean (SD)	23.7 (3.8)
Daily use of vitamin D-containing butter or margarine on bread or in cooking, n (%)	25 (45.5)
Regular consumption of vitamin D enriched milk, n (%)	14 (25.5)
Intake of fatty fish at least twice a week, n (%)	32 (58.2)
Habitual use of dietary supplements other than vitamin D, n (%)	7 (12.7)
Common use of sunscreen on sunny days, n (%)	18 (32.7)
Exposes skin to direct sunlight > 10 minutes on sunny days, n (%)	49 (89.1)
Spends on average more than 2 hours outdoors during a sunny week, n (%)	36 (65.5)

### Effect of supplementation on vitamin D status

Mean (SD) s-25(OH)D in the sample increased from 44.3 (23.6) nmol/l to 78.4 (24.5) nmol/l, with a mean (SD) increase (Δ25(OH)D) of 34.1 (13.1) nmol/l (Table 2). Individual Δ25(OH)D ranged from −12 nmol/l to 58 nmol/l. Δ25(OH)D did not differ by type of supplement, as previously published [14]. At baseline, one person had s-25(OH)D <12.5 nmol/l and 13 persons (24%) had s-25(OH)D < 25 nmol/l. Four persons (7%) had s-25(OH)D > 75 nmol/l. At follow-up, none had s-25(OH)D <25 nmol/l. Five participants (9%) had s-25(OH)D <50 nmol/l. Twenty-nine participants (53%) had s-25(OH)D >75 nmol/l at follow-up.

**Table 2:** Mean (SD) unadjusted serum levels of markers of the vitamin D endocrine system and bone turnover at baseline and follow-up according to type of supplement

***Variable***	***n***	***Mean (SD) serum level at baseline***	***Mean (SD) serum level at follow-up***	***Mean (SD) change during intervention***	***p value*^*(2)*^**
**s-25(OH)D (nmol/l)**					
Overall	55	44.3 (23.6)	78.4 (24.5)	34.1 (13.1)	<0.001
Fish oil capsules	27	48.5 (24.8)	80.4 (25.0)	31.9 (15.3)	<0.001
Multivitamin tablets	28	40.3 (22.0)	76.5 (24.3)	36.2 (10.4)	<0.001
p value ^(1)^		0.20	0.56	0.22	
**s-iPTH (pmol/l)**					
Overall	55	5.9 (2.6)	4.7 (2.1)	−1.2 (2.5)	0.001
Fish oil capsules	27	5.7 (2.6)	4.9 (2.5)	−0.9 (2.8)	0.13
Multivitamin tablets	28	6.0 (2.7)	4.5 (1.6)	−1.4 (2.2)	0.002
p value ^(1)^		0.77	0.52	0.40	
**s-1,25(OH)**_**2**_**D (pmol/l)**					
Overall	53	121 (41)	134 (38)	13 (48)	0.057
Fish oil capsules	27	128 (38)	136 (34)	8 (45)	0.37
Multivitamin tablets	26	113 (43)	131 (42)	18 (50)	0.09
p value ^(1)^		0.20	0.64	0.46	
**s-TRACP (U/l)**					
Overall	54	2.65 (0.70)	3.03 (0.69)	0.38 (0.33)	<0.001
Fish oil capsules	27	2.59 (0.80)	3.01 (0.77)	0.43 (0.31)	<0.001
Multivitamin tablets	27	2.71 (0.61)	3.04 (0.60)	0.34 (0.36)	<0.001
p value ^(1)^		0.53	0.87	0.32	

^(1)^ Differences between types of supplement are tested using *t* test.

^(2)^ Paired-samples *t* test for comparison of individual pre- and post-intervention concentration.

### Effect of supplementation on PTH and TRACP

S-iPTH decreased by mean 1.2 pmol/l, from 5.9 pmol/l to 4.7 pmol/l (Table 2), and the decrease did not differ significantly by type of supplement. The decrease was significant in the multivitamin group where s-iPTH at baseline was accidentally higher. There was an overall borderline statistically significant increase in s-1,25(OH)_2_D. Individual Δs-1,25(OH)_2_D showed a large variation, from −135 to 120 pmol/l. S-TRACP increased significantly from mean 2.65 to mean 3.03 U/L during supplementation.

### Potential predictors of effect of supplementation

**Figure 1:**
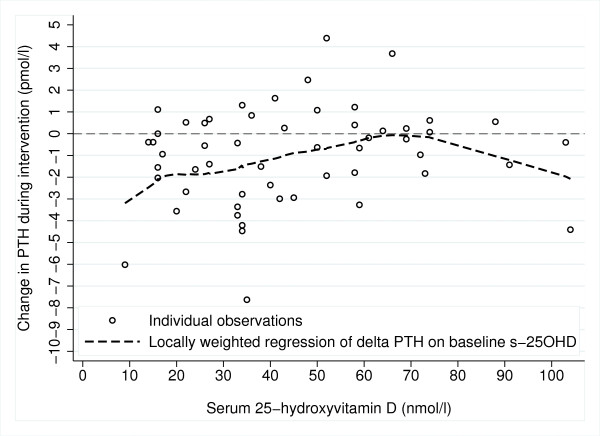
Relationship between vitamin D status at baseline and change in PTH (n = 55).

Results of linear regression analysis are shown in Table 3. Baseline s-25(OH)D was predicted only by ethnic background, Norwegians having unadjusted mean 33 (95% CI 20, 46) nmol/l higher baseline s-25(OH)D than Tamils. However, ethnic background did not affect increase in vitamin D status. Mean (SD) increase in s-25(OH)D was 33.8 (14.1) in Norwegians and 36.2 (10.0) in Tamils, p = 0.60. No background characteristics were significantly associated with increase in s-25(OH)D during supplementation (Table 3). The changes in the biochemical parameters (except 25(OH)D) were inversely associated with their baseline concentrations. Decrease in s-iPTH during supplementation tended to be larger when baseline s-25(OH)D was lower (Figure 1).

**Table 3:** Predictors of change in biochemical parameters during four weeks of vitamin D3 supplementation (n = 55)

**Dependent variable**	**Δs-25(OH)D (nmol/l)**		**Δs-iPTH (pmol/l)**		**Δs-1,25(OH)**_**2**_**D (pmol/l)**		**Δs-TRACP (U/L)**	
	**B** **(95% CI)**	***p value***	**B** **(95% CI)**	***p value***	**B** **(95% CI)**	***p value***	**B** **(95% CI)**	***p value***
Type of supplement (solid multivitamin tablet vs. gelatine fish oil capsule with n-3 fatty acids) ^(1)^	3.5 ^(2)^ (−3.6, 10.6)	0.33	−0.4 (−1.5, 0.6)	0.39	−1 (−22, 19)	0.89	−0.08 (−0.25, 0.10)	0.40
Age, per 10 years	1.4 (−3.2, 6.0)	0.54	−0.5 (−1.3, 0.4)	0.31	−10 (−27, 7)	0.24	0.07 (−0.05, 0.19)	0.25
Gender, men vs. women	1.0 (−6.4, 8.5)	0.78	−1.3 (−2.5, 0.3)	0.11	18 (−8, 46)	0.17	0.04 (−0.16, 0.23)	0.71
Body mass index, per 5 kg/m2	−1.0 (−5.7, 3.7)	0.68	−0.3 (−1.2, 0.6)	0.48	−9 (−26, 8)	0.30	0.05 (−0.07, 0.17)	0.41
Ethnic background, Tamil vs. Norwegian	2.7 (−6.0, 11.3)	0.54	−0.5 (−2.2, 1.2)	0.54	−18 (−51, −14)	0.26	0.27 (0.05, 0.49)	0.016
Baseline s-25(OH)D, per 10 nmol/l	−1.1 (−2.6, 0.4)	0.13	0.2 (−0.1, 0.5)	0.10	−3 (−8, 3)	0.31	−0.03 (−0.07, 0.01)	0.10
Baseline s-iPTH, per 5 pmol/l	4.4 (−2.3, 11.2)	0.20	−3.2 (−4.2, −2.3)	< 0.001	−13 (−38, 12)	0.30	−0.04 (−0.22, 0.14)	0.66
Baseline s-1,25(OH)_2_D, per 10 pmol/l	−0.3 (−1.2, 0.6)	0.51	0.0 (−0.2, 0.1)	0.71	−8 (−10, −5)	< 0.001	< −0.01 (−0.03, 0.02)	0.81
Baseline s-TRACP, per U/l	−4.7 (−9.8, 0.3)	0.07	0.1 (−0.9, 1.1)	0.86	9 (−10, 28)	0.34	−0.13 (−0.26, -0.01)	0.039

^1^ Groups are randomised with regard to type of supplement. The analysis of type of supplement is adjusted for baseline serum concentration of the respective dependent variables, while the other analyses in the table are unadjusted.

^2^ Result previously published in: Holvik K *et al.*, Br J Nutr 2007; 98: 620–5.

There was a significant association between ethnic background and ΔTRACP, Tamils having higher increase in s-TRACP than Norwegians (Table 3). This was not significant when adjusting for the lower baseline s-TRACP in Tamils (p = 0.07).

There was no effect of type of supplement (fish oil capsule or multivitamin tablet) on change in any biochemical parameter.

## Discussion

We found that supplementation with 10 μg vitamin D3/day administered for four weeks in late winter led to vitamin D sufficiency (s-25(OH)D > 50 nmol/l) in healthy adults of ethnic Norwegian background, but not completely so in Tamils living in Oslo (59°N). This was due to a very low baseline vitamin D status in Tamils, which we have also observed previously [15]. In spite of the strict exclusion criteria, vitamin D status at baseline was not very low in ethnic Norwegian participants. A large proportion of the study sample were medical and nursing students, probably more health-conscious than the general population, and may have had a relatively high intake of vitamin D intake during winter. However, the supplementation was equally efficient regardless of ethnic background, and led to an overall mean increase in s-25(OH)D of 34.1 nmol/l in the course of four weeks. We found no effect of type of vitamin D supplement (solid multivitamin tablet or fish oil capsule containing n-3 fatty acids) on either increase in vitamin D status [14] or in the change in parathyroid hormone, the active vitamin D hormone, or osteoclast activity. As far as we are aware, this is the first randomised trial to compare the effect of two different modes of administration of vitamin D3 on markers of the vitamin D endocrine system and bone turnover.

S-25(OH)D above 50 nmol/l has been associated with optimal musculoskeletal function in elderly [16], and this level is commonly used as cut-off for vitamin D sufficiency in European populations [17-19]. However, some advocate a target s-25(OH)D in the adult European and US population of at least 75 nmol/l for optimal fall and fracture prevention, and this requires daily supplementation of at least 18–25 μg (700–1000 IU) [20]. However, few intervention studies have focused on supplementation in young adult individuals and its influence on markers of bone health.

Unlike other intervention studies with a similar dose of vitamin D [8,11-13], we observed a significant suppression of PTH as well as increased bone turnover in our study.

Vitamin D supplementation is expected to prevent bone loss mainly by slowing bone resorption. Due to the short follow-up time, we expected any effect on bone turnover likely to be observed in resorption rather than formation. The TRACP isoform 5b is released by osteoclasts early in their differentiation, and this enzyme is specifically a marker of number of osteoclasts rather than of their resorptive activity. In healthy individuals, however, the osteoclast number and their resorptive activity is expected to be highly correlated [21]. TRACP 5b was also studied in the recent Finnish supplementation study in healthy men aged 21–49 years (n = 48) [8]. The investigators observed a decrease in this marker throughout winter (6 months) regardless of whether the participants received placebo, 10, or 20 μg vitamin D. The bone formation marker (bone alkaline phosphatase) decreased in the groups receiving vitamin D supplementation but was unaltered in the placebo group.

The suggested increase in osteoclast activity in our data in spite of the improvement in vitamin D status and corresponding PTH suppression is surprising, but may represent a transient high turnover state with increased bone turnover as a result of increased bone formation. The participants were relatively young, and 40% were below 25 years of age. Peak bone mass may not have been acquired in all participants [22]. An increased bone turnover may thus suggest an improved rate of bone growth in young adults. As bone formation and resorption are coupled processes, increased formation will follow the increase in resorption. A decrease in PTH would be expected to suppress bone resorption, but bone resorption is also stimulated by 1,25(OH)_2_D. Local production of 1,25(OH)_2_D in the tissues may have increased, while we have only measured circulating 1,25(OH)_2_D. There is evidence that 25(OH)D affects osteoclastogenesis and bone turnover (and thereby TRACP production) by local activation in bone cells [23]. In a recent review, it was considered that due to this local activation, circulating levels of 25(OH)D, and not 1,25(OH)_2_D, may represent the better correlate to parameters of bone health [24].

There is ongoing research concerning the role of n-3 fatty acids in bone health, as bone loss due to increased osteoclast activity may be mediated by inflammatory cytokines, and some studies suggest a protective effect of n-3 fatty acids on bone resorption [25,26]. In bone marrow from ovarioectomized mice, addition of eicosapentaenoic acid and docosahexaenoic acid led to significantly reduced TRACP activity and osteoclastogenesis [27]. A protective effect of n-3 fatty acids (0.5 g/day) in fish oil on bone resorption was not supported in our data in young adults, as subjects’ s-TRACP increased regardless of being randomised to ingest fish oil capsules containing omega-3 fatty acids, or solid tablets. The increase in s-TRACP was even slightly higher in the group ingesting fish oil capsules (not shown/not significant).

It cannot be excluded that an independent effect of the supplementation on bone turnover regardless of the observed decrease in PTH could be brought about by factors other than vitamin D. Both intervention supplements contained vitamin A (retinol). Although the evidence of an effect of vitamin A on bone health is inconsistent, some in vitro studies have shown that retinoic acid directly stimulate osteoclastic bone resorption, and high intakes and serum levels of retinol have been associated with reduced bone mineral density or increased fracture risk in some population-based studies [28].

Our study was a parallel-group intervention and we did not include a placebo group. Due to the late wintertime intervention period we did not expect any notable background increase in vitamin D status. Moreover, any background change in vitamin D status would be the consequence of any unexpected behaviour in relation to diet or sun exposure in both parallel-groups regardless of supplement. Our study sample was restricted to not take a regular supplement other than the intervention supplement, use a tanning bed, or travel to sunny areas. Any extraordinary dietary intake (e.g. large fatty fish dinner) would be noted on the participants’ individual compliance form, and no such behaviour was reported. Also, only those who were compliant defined as consuming at least 26 of the 28 tablets were included in the analyses. Thus, there is reason to expect that any observed change in the vitamin D endocrine system is an effect of the supplementation.

A limitation of the ethnic comparison was the different age distributions between participants with ethnic Norwegian and Sri Lankan Tamil background. Mean age in the two groups differed by 14 years. While more than half of the subjects with ethnic Norwegian background were below 30 years of age, none of those with Sri Lankan Tamil background were below that age. Age is an important predictor of serum PTH [10], but limited overlap in age between the ethnic groups in our study makes it difficult to rule out the influence of age and ethnic background, respectively.

## Conclusions

We conclude that four weeks of daily supplementation with 10 μg (400 IU) vitamin D3 to healthy adults during wintertime led to a decrease in s-iPTH and an increase in circulating TRACP. These effects did not differ by mode of administration. In spite of different serum levels at baseline in Norwegians and Tamils, the effect was similar in the two ethnic groups.

## Ethical approval

The study protocol was reviewed by the Regional Committee for Medical Research Ethics and approved by the Norwegian Data Inspectorate. Written informed consent was collected from all participants.

## Competing interests

One of the intervention supplements used was provided freely for this research project by the manufacturer (Möller's). We declare that the Authors have no employment or personal financial interest in relation to the work described.

## Authors’ contribution

KH and AAM planned and carried out the data collection under guidance of HEM and LCS. AAM prepared the data file. KH analysed the data and wrote the manuscript. HEM and LCS initiated the study and contributed to the methods and the design of the paper. CML was responsible for the serum sample analyses. All co-authors have critically revised and approved the manuscript.

## Pre-publication history

The pre-publication history for this paper can be accessed here:

http://www.biomedcentral.com/1472-6823/12/7/prepub
